# Ecological Analysis of the Helminth Community of *Microtus lusitanicus* (Gerbe, 1879) (Rodentia) in Asturias (NW Spain)

**DOI:** 10.3390/ani11113055

**Published:** 2021-10-26

**Authors:** Roser Adalid, Carles Feliu, Aitor Somoano, Marcos Miñarro, Jacint Ventura, Jordi Torres, Jordi Miquel, Màrius Vicent Fuentes

**Affiliations:** 1 Secció de Parasitologia, Departament de Biologia, Sanitat i Medi Ambient, Facultat de Farmàcia i Ciències de l’Alimentació, Universitat de Barcelona, Av. Joan XXIII, sn, 08028 Barcelona, Spain; roseradalid@ub.edu (R.A.); cfeliu@ub.edu (C.F.); jtorres@ub.edu (J.T.); jordimiquel@ub.edu (J.M.); 2 Institut de Recerca de la Biodiversitat (IRBio), Universitat de Barcelona, Av. Diagonal, 645, 08028 Barcelona, Spain; 3 Servicio Regional de Investigación y Desarrollo Agroalimentario (SERIDA), Ctra. AS-267, PK 19, 33300 Villaviciosa, Spain; aitors@serida.org (A.S.); mminarro@serida.org (M.M.); 4 Departament de Biologia Animal, Biologia Vegetal i Ecologia, Facultat de Biociències, Campus de Bellaterra, Universitat Autònoma de Barcelona, 08193 Cerdanyola del Vallès, Spain; jacint.ventura.queija@uab.cat; 5 Àrea de Recerca en Petits Mamífers, Museu de Ciències Naturals de Granollers “La Tela”, C/Palaudàries, 102, 08402 Granollers, Spain; 6 Parasites and Health Research Group, Departament de Farmàcia i Tecnologia Farmacèutica i Parasitologia, Facultat de Farmàcia, Universitat de Valencia, Av. Vicent Andrés Estellés, 46100 Burjassot, Spain

**Keywords:** helminth community, Lusitanian pine vole, *Microtus lusitanicus*, Asturias, Spain

## Abstract

**Simple Summary:**

The Lusitanian pine vole is an endemic rodent of the Iberian Peninsula, which has a burrowing behaviour and prefers to live underground. It feeds on bark and roots causing severe damage to trees. In Asturias (NW Spain), this species is considered a pest causing economic losses in apple orchards, damaging the tree, and sometimes even causing its death. With the aim to shed light on the helminth community of this rodent pest species and to elucidate which intrinsic and extrinsic factors affect its helminth species, a faunistic-ecological study was carried out. For this purpose, our own collection of 710 voles from several orchards of various locations in Asturias was used. The results of the ecological study revealed the influence of climate variables, the year and season of capture, as well as the host age, on the diversity of the helminth community and the infection parameters of helminth species, underlining the importance of their life cycles. Our findings on the helminth community of the Lusitanian pine vole in Asturias could be used to improve the biological methods applied to control the population of this rodent pest.

**Abstract:**

The Lusitanian pine vole, *Microtus lusitanicus*, an endemic fossorial rodent of the Iberian Peninsula, has a burrowing behaviour and prefers to live underground. It feeds on bark and roots causing severe damage to trees. In Asturias (NW Spain), where *M. lusitanicus* is considered a pest in several orchards, a faunistic-ecological study was carried out to describe the helminth community of this species and the main factors that could influence its helminth component species. For this purpose, our own collection of 710 voles from several orchards of various locations in Asturias was used. Eight helminth species, four cestodes and four nematodes, were found. Statistical non-parametric tests were used to analyse the effects of extrinsic and intrinsic factors on the diversity of the helminth community and species prevalence and abundance. The results show the influence of climate variables, the year and season of capture, as well as host age, on the diversity of the helminth community and the infection parameters of some helminth species, underlining the importance of their life cycles. In addition to shedding light on the helminth community of this rodent in Asturias, the results obtained could be used to improve the biological methods applied to fight the *M. lusitanicus* pest.

## 1. Introduction

The Lusitanian pine vole, *Microtus lusitanicus* (Gerbe, 1879), is an endemic fossorial rodent, which inhabits the northwestern quadrant of the Iberian Peninsula and the southwestern tip of France [1]. It can be found from sea level to up to 2000 m, in both natural and agricultural habitats [1,2]. In all cases, the ground must be soft and humid with a high vegetal cover, which are the necessary conditions for the voles to build their gallery systems [1]. This rodent has a burrowing behaviour and prefers to live underground, although surface movements under abundant vegetation may be frequent. Vole galleries remain open as a small hole in the ground [3] with superficial galleries used for feeding and escaping and deep ones used for storing food and nesting [1]. *Microtus lusitanicus* is a monogamous species: a couple of voles and their offspring (juvenile and sub-adult individuals) share the same gallery system [4,5,6]. Lusitanian pine voles are small sized rodents (body length: 77.5–105.0 mm; body mass; 14.0–19.0 g) with big head, blunt snout, small eyes and cylindrical body [1].

*Microtus lusitanicus* can cause severe damage to fruit trees, carrots or potatoes [1]. In Asturias (northwest Spain), this rodent species is, along with the montane water vole, *Arvicola scherman* (Shaw, 1801), one of the main causes of economic losses in apple orchards [3,7,8]. Both species feed on bark and roots, damaging the tree, and sometimes even causing its death [3]. In Spain, *M. lusitanicus* is officially considered a pest, and the control of its populations is recommended [9]. Nowadays, rodenticides should comply with current legislation and not involve adverse environmental consequences, produce resistance in the target species, unnecessary pain or to be a threat to non-target animals and humans (EU Regulation 528/2012) [10], making the examination of other potential strategies necessary. Among specific and environmentally benign control practices, increasing landscape heterogeneity, fertility control, the use of repellents or biocontrol (predators, parasites and other pathogens) have been proposed as promising tools [11,12].

Parasites may have a negative effect on host fitness [13]. Host–parasite models predict that parasites can regulate the population dynamics of the hosts [14,15]. Regulation is the process through which high population numbers are lowered to a normal level, but also increased up to this level when they reach low values [16]. The aim of pest control is to reduce the population size to lower levels than the natural equilibrium. It must be considered that many pest species cause damage in spite of their low population densities. To reach this goal, it is necessary that the negative impact of the parasite on the host must exceed the population’s intrinsic growth rate and infection, and that this impact needs to be persistent. Parasites can play two roles in rodent pest management: as bio-rodenticides or as vehicles of immunocontraception causing sterility to infected animals [17].

The helminth fauna of *M. lusitanicus* in the Iberian Peninsula was previously analysed [18,19]. These studies, carried out on voles from “damaged vegetable gardens” in several localities of the Iberian Peninsula, especially in the central-western part, reported the detection of a total of 14 helminth species: 1 trematode, 7 cestodes, and 6 nematodes. The structure and dynamics of the vole’s helminth community and the environmental factors influencing it have also been analysed. Environmental factors, such as temperature and rainfall, as well as intrinsic factors, such as host age and sex, were found to influence the helminth community of the Lusitanian pine vole.

The main aims of the present study are to shed light on the helminth community of *M. lusitanicus*, to elucidate the potential effect extrinsic and intrinsic factors have on it, and to update and expand previously reported data. Moreover, some new proposed hypotheses could be confirmed: (1) pesticides used in pest control in areas of intensive agricultural use are responsible for the absence of some helminth species found in other localities belonging to the geographical distribution of the Lusitanian pine vole, due to the loss of invertebrate intermediate hosts; (2) the parasitological patterns of those helminth species that are influenced by climate variables will be affected by global warming and/or climate change; (3) the results can be used to improve the biological control methods against this vole pest in agro-ecosystems.

## 2. Materials and Methods

**Figure 1:**
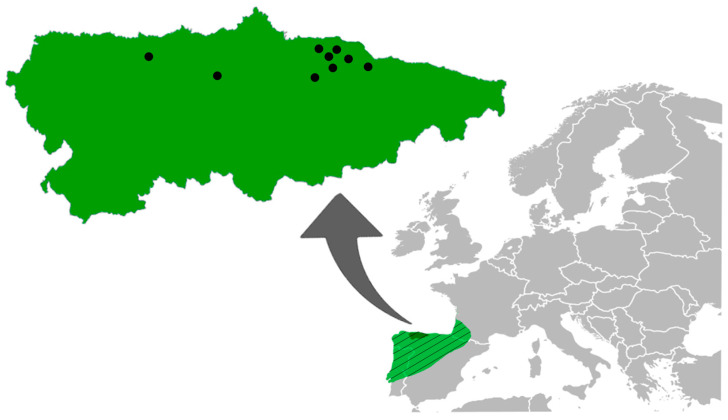
Distribution of *Microtus lusitanicus* in the Iberian Peninsula (**striped green**) and map of Asturias (**dark green**) showing the trapping sites of the individuals analysed (**black points**).

Asturias is located on the northern coast of Spain (43°30′ N, 5°30′ W) (Figure 1). The climate is temperate oceanic with abundant rainfall spread fairly throughout the year and mild temperatures both in winter and summer. This area is characterized by small agricultural plots separated by hedgerows and woodlands, with an irregular topography of smooth hills and valleys. The fertile soil added to these weather conditions favours the establishment of an evergreen and dense vegetal cover in orchards all year round [3,20,21].

### 2.1. Zoological and Helminthological Procedures

**Figure 2:**
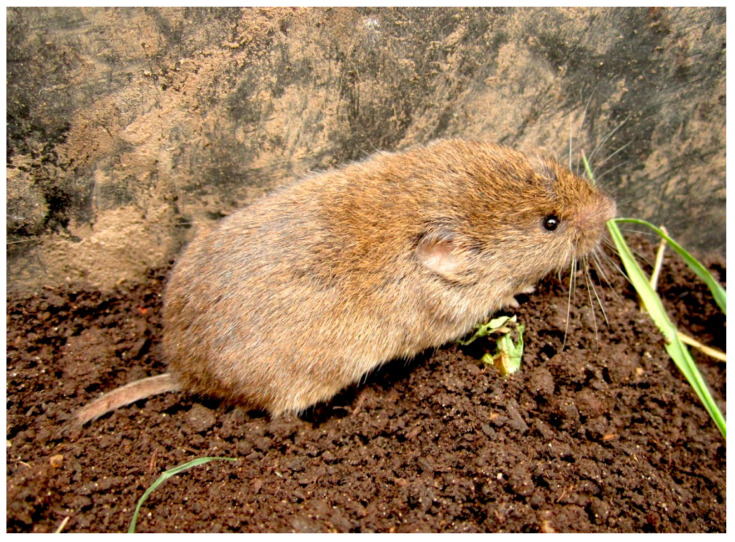
An individual of *Microtus lusitanicus* in its natural habitat.

A total of 710 *M. lusitanicus* (Figure 2) individuals obtained from the collection of SERIDA (Servicio Regional de Investigación y Desarrollo Agroalimentario, Asturias) was helminthologically analysed. The sample was collected during a population peak, for two consecutive years (January 2011 to January 2013), in apple orchards of 9 Asturian localities (Figure 1). The recommendations of the Directive of the European Parliament and of the Council on the Protection of Animals Used for Scientific Purposes [22] were followed in the field work.

Of the total individuals analysed, 343 were captured in 2011, 354 in 2012 and 13 in 2013. Considering sex, 363 were male and 347 were female. Animals were classified by body mass in three age groups: juveniles, ≤10 g; sub-adults 10.1–14.9 g: adults, ≥15 g [2]. According to this criterion, 63 specimens were juveniles, 334 were sub-adults and 313 were adults. Seasons were defined as: winter (January–March), spring (April–June), summer (July–September) and autumn (October–December). Following this distribution by months, 139 captures took place in winter, 187 in spring, 196 in summer and 188 in autumn. A comprehensive description of the host sample analysed is shown in Table 1 and Table 2.

In the laboratory, specimens were dissected, and all viscera were extracted for the parasitological study. All helminths found were preserved in ethanol 70%. Cestodes were stained with alcoholic chlorhydric carmine, differentiated with acidified ethanol, dehydrated in alcohol series, cleared with xylene and mounted in Canada balsam. Some cestode scolices and some nematodes were cleared in Amann lactophenol. The helminth specimens were identified at specific level, based on their morphology and morphometry according to the most relevant descriptions and findings in the scientific literature.

### 2.2. Helminth Community Analysis

The helminth community composition and structure of the Lusitanian pine vole were analysed considering each particular life cycle as well as prevalence, mean abundance, range and total number of helminths calculated [23].

The analysis of helminth community components was made by means of calculating the frequency of occurrence of the number of helminth species, the abundance index and the frequency distribution of helminths. The abundance index (AI) (excluding those parasite species for which *M. lusitanicus* acts as an intermediate host) was calculated [24,25]. The helminth community of a host species is characterized by the presence of the following categories of species: dominant species (AI > 1); co-dominant species (0.1 ≤ AI ≤ 1); successful immigrant species (0 < AI < 0.1); unsuccessful immigrant species (AI = 0).

The frequency distribution of helminth species was calculated by means of the Lefkovitch index (L) [26], where

L = (1/45)tang^−1^(variance/mean) − 1 (1)

ranging from −1 (positive binomial or uniform distribution), 0 (Poisson or random distribution) to +1 (negative binomial or aggregated distribution).

The diversity/uniformity analysis of the helminth community was carried out using the Shannon index (H’) [27,28], Simpson index (D’) [29] expressed as 1-D’ [28], Berger-Parker index (d) [30,31], expressed as 1-d [28] and Shannon evenness index (E) [28,32].

The helminth infracommunity structure study was established using the analysis of the number of helminths, number of helminth species, the Brillouin index (HB) [27,28], Brillouin index for infected hosts only and percentage of infected hosts.

The role played by intrinsic (host sex and age) and extrinsic (climate data, year and season of capture) factors (independent variables) in determining the helminth community dependent variables (species richness − the number of helminth species; the helminth community diversity—Brillouin index; the prevalence—% of parasitation; and the worm burden − helminth abundance) was statistically analysed. Climate data, related to temperature (mean daily temperature calculated for each season) and precipitation (mean daily rainfall calculated for each season and cumulative rainfall during each season) (Table 3), were obtained from the nearest climate stations (AEMET—Spanish Meteorological Agency). Values of climate variables, belonging to the year before capture (which are related to both the helminth and vole life cycles), were correlated with the annual values of the dependent variables by means of the Spearman correlation coefficient (*r*_s_); prevalence was previously transformed logarithmically, log (x/(1 − x)). The influence of year and season of capture, and host sex and age on prevalence was analysed using binary logistic regression (BLR), while their influence on the other dependent variables was analysed by means of standard non-parametric tests, i.e., the Mann–Whitney (U) and Kruskal–Wallis (H) tests.

SPSS 26.0—IBM for Windows was the software package used for statistical analysis. Statistical significance was established at *p* < 0.05.

## 3. Results

### 3.1. Helminth Species

A total of 594 Lusitanian pine voles (83.67%) were found to be infected with a total of eight helminth species: four cestodes and four nematodes (Table 4). Among the helminth species observed, although the hymenolepidid *Rodentolepis asymmetrica* and the heligmosomid *Carolinensis minutus* have been previously reported in other Arvicolinae host species [18], both helminth species are now reported for the first time parasitizing *M. lusitanicus*.

The morphology and morphometry of some stages of the helminth specimens found parasitizing the voles were analysed under the microscope and some specific measurements were made and compared with those reported in descriptions of the scientific literature, making it possible to identify all of them at specific level, with the only exception being a cysticercoid found in the intestine of a vole.

#### 3.1.1. *Hydatigera taeniaeformis* (Batsch, 1786) Larvae

**Figure 3:**
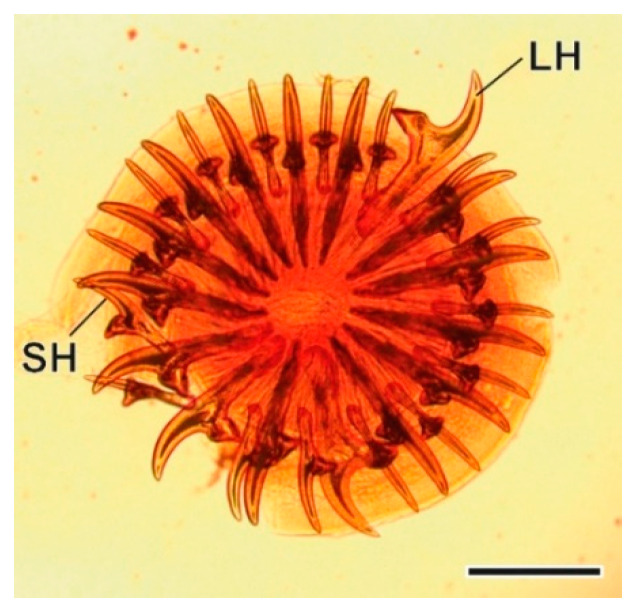
Rostellar hooks of *Hydatigera taeniaeformis* larvae. LH, large hook; SH, small hook. Scale bar = 300 µm.

**Figure 4:**
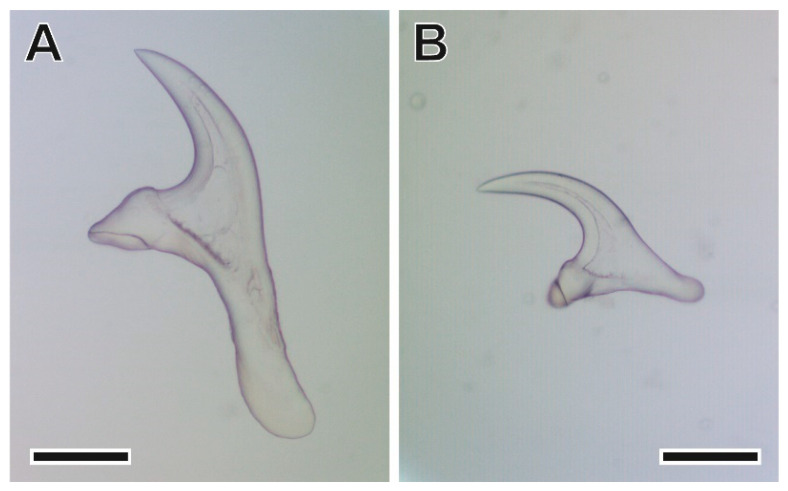
Large (**A**) and small (**B**) hooks of *Hydatigera taeniaeformis* larvae. Scale bar = 200 µm.

The larval stage of this taenid tapeworm, a strobilocercoid englobed in a cyst of a 5–12 mm diameter, was found on the liver. The scolex (Figure 3 and Figure 4) has a rostellum with 30–36 hooks of two different sizes [33].

#### 3.1.2. *Paranoplocephala omphalodes* (Hermann, 1783)

This anoplocephalan tapeworm was found in the small intestine. The unarmed scolex, the morphology and disposition of reproductive organs, as well as the size and morphology of the eggs obtained from gravid proglottids allow its identification at specific level [34].

#### 3.1.3. *Rodentolepis asymmetrica* (Janicki, 1904)

The adult stage of this hymenolepidid cestode was found in the small intestine. The armed scolex has a rostellum with 18–21 hooks of 18–20 μm. The eggs are spherical, with a diameter of 45–67 μm, containing an oncosphere of a 20–30 μm diameter, with embryonic hooks of 8.5–9 μm length [34].

#### 3.1.4. *Trichuris arvicolae* Feliu et al., 2000

**Figure 5:**
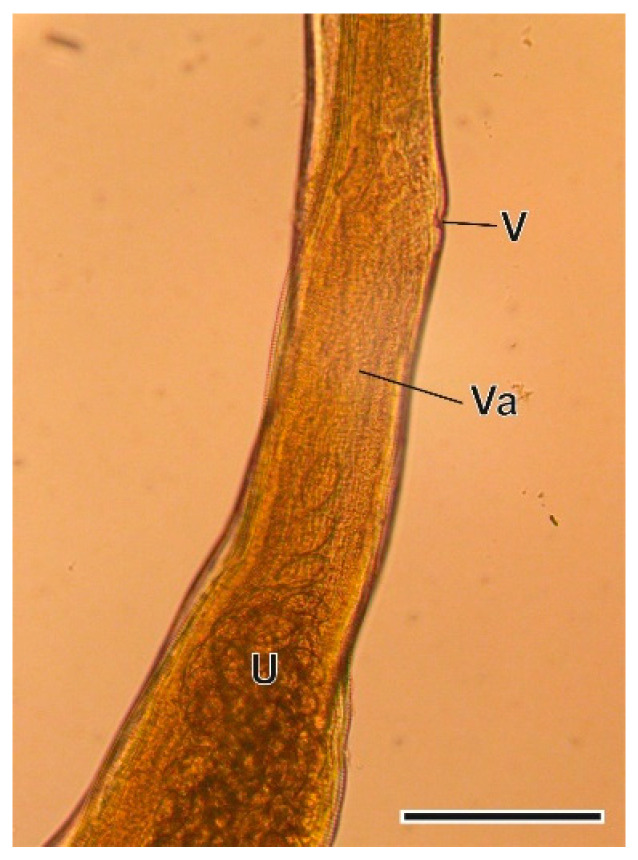
Vulvar region of a *Trichuris arvicolae* female. U, uterus; V, vulva; Va, vagina. Scale bar = 200 µm.

**Figure 6:**
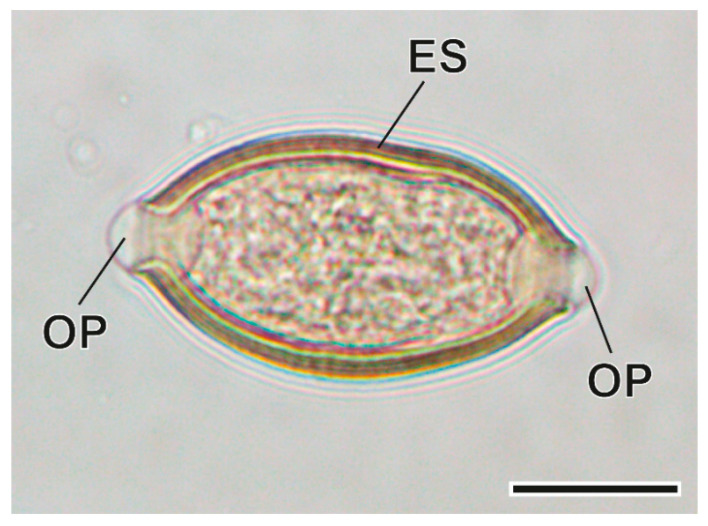
Egg of *Trichuris arvicolae*. ES, eggshell; OP, opercular plugs. Scale bar = 25 µm.

This Trichurinae nematode was found in the caecum. The morphology of the oesophagus, as well as the morphology and morphometry of female and male sexual organs and eggs (Figure 5 and Figure 6) allowed the recent description of this nematode as a new species [35].

#### 3.1.5. *Carolinensis minutus* (Dujardin, 1845)

**Figure 7:**
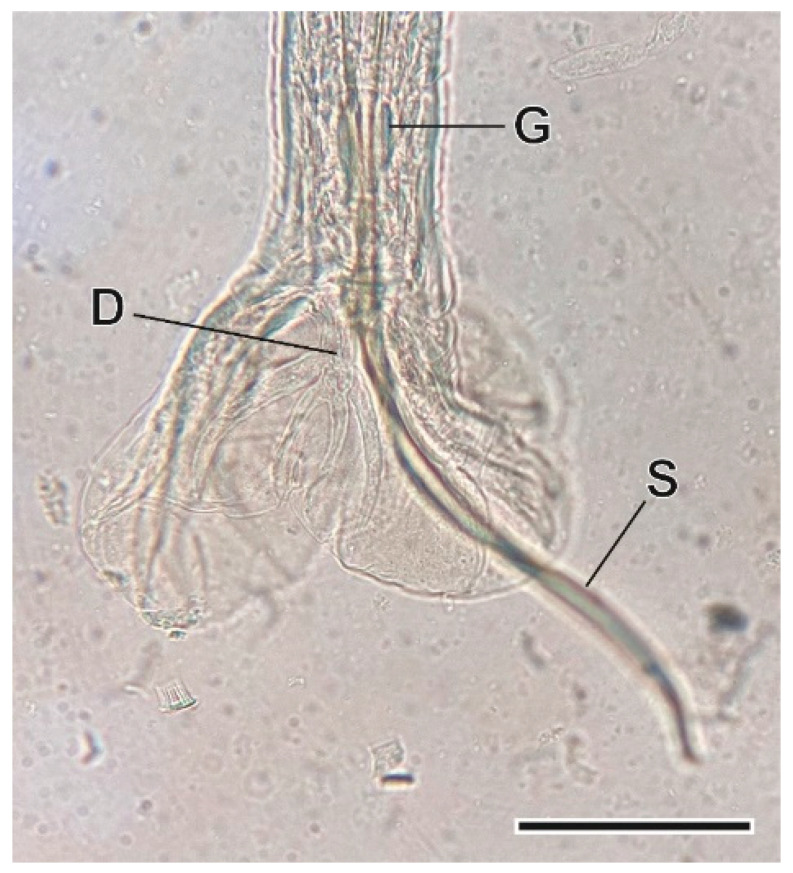
Caudal bursa of a *Carolinensis minutus* male. D, dorsal ray; G, gubernaculum; S, spicules. Scale bar = 1 µm.

This heligmosomid nematode was found in the small intestine. It is characterized by its very small size, the presence of longitudinal ridges that cover its body, the vesicle that covers its head, and the caudal bursa of males [36] (Figure 7).

#### 3.1.6. *Heligmosomum costellatum* (Dujardin, 1845)

**Figure 8:**
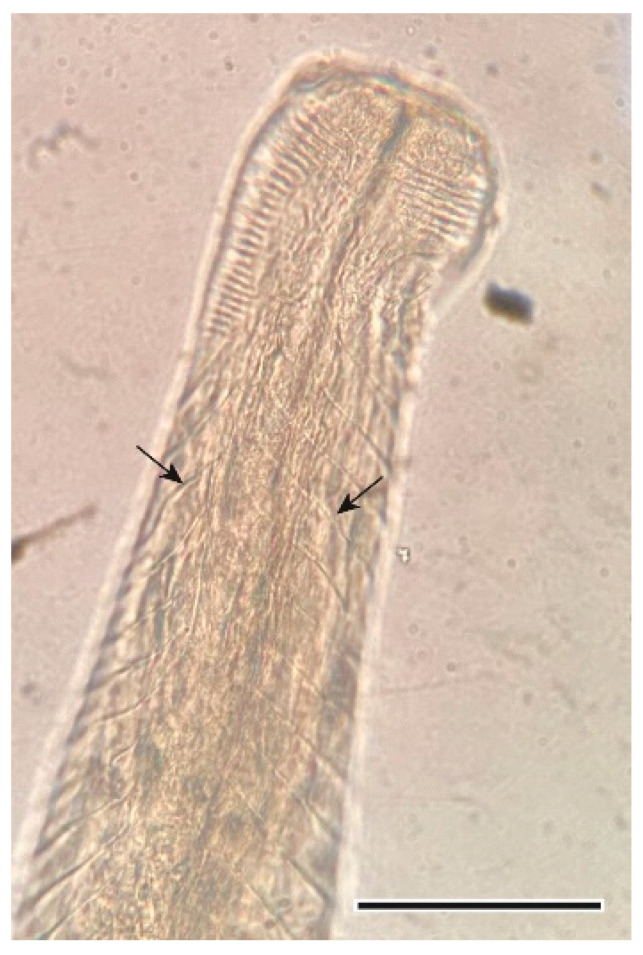
Cephalic extremity of a *Heligmosomum costellatum* female. Arrows point at cuticular striations. Scale bar = 100 µm.

**Figure 9:**
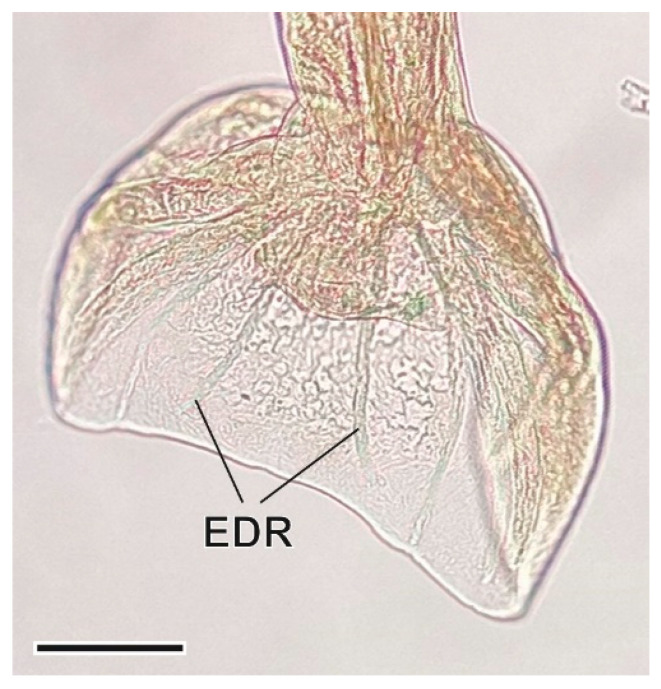
Caudal bursa of a *Heligmosomum costellatum* male. EDR, externodorsal rays. Scale bar = 100 µm.

This other heligmosomid nematode was found in the small intestine. It presents transversal stretch marks that cover its cuticle, and a typical caudal bursa of males [36] (Figure 8 and Figure 9).

#### 3.1.7. *Syphacia nigeriana* Baylis, 1928

**Figure 10:**
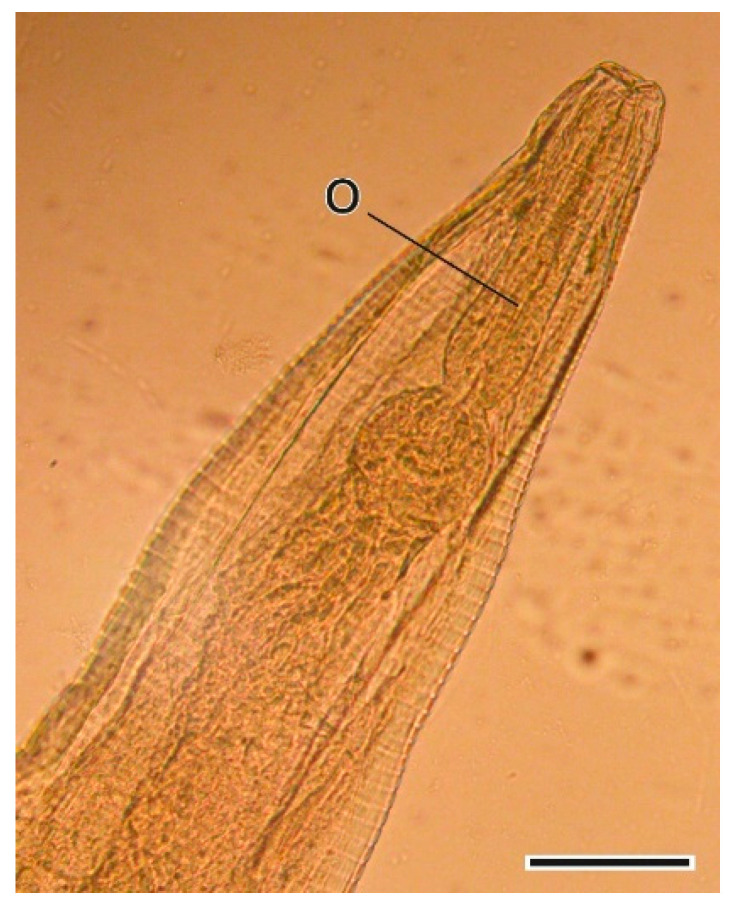
Cephalic extremity of a *Syphacia nigeriana* female. O, oesophagus. Scale bar = 100 µm.

**Figure 11:**
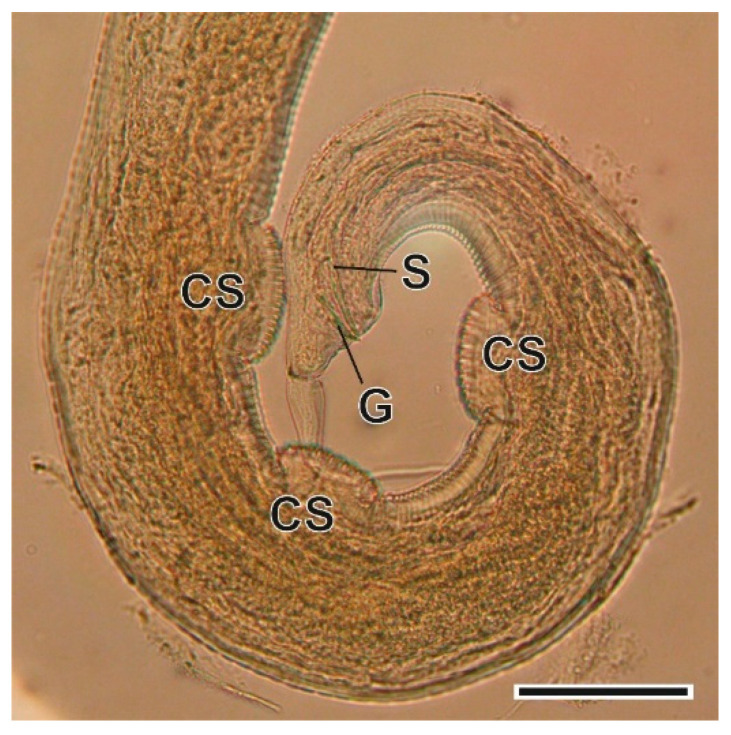
Caudal extremity of a *Syphacia nigeriana* male. CS, ventral cuticular swellings; G, gubernaculum; S, spicule. Scale bar = 100 µm.

This oxyurid nematode was found in the caecum. The oxyuriform oesophagus is one of the most relevant characteristics at genus level. The morphological and morphometric characterization of its sexual organs is necessary to enable identification at species level [36] (Figure 10 and Figure 11).

### 3.2. Helminth Community Analysis

*Carolinensis minutus* showed the highest prevalence of infection (43.38%) and was the most abundant (5.80), while *S. nigeriana* presented the highest mean intensity value (5). The five most prevalent species, *C. minutus* (43.38%), *S. nigeriana* (22.25%), *H. taeniaeformis* larvae (8.31%), *T. arvicolae* (5.07%) and *H. costellatum* (3.52%), were analysed considering sex, age, season and year of capture of the host (Table 5 and Table 6).

More than 60% of the hosts presented either one or two helminth species, but infracommunities of up to 4 species were also found (Table 7). The abundance index values (Table 8) make it possible to establish the following community structure: *C. minutus* and *S. nigeriana* as dominant species; *H. costellatum* as co-dominant species; and *T. arvicolae* as successful immigrant species. *Paranoplocephala omphalodes* and *R. asymmetrica* are considered as non-successful immigrant species. The two larval stages (*H. taeniaeformis* and *Cysticercoid* sp.) are not included in this classification since the Lusitanian pine vole acts as intermediate host.

The frequency distribution of the most prevalent helminth species (Table 8) showed that all species analysed presented a negative binomial distribution, but *H. taeniaeformis* and *T. arvicolae* were close to a Poisson or random distribution.

The values of the Shannon, Simpson, Berger–Parker and Shannon evenness indices reflect the diversity/uniformity of the helminth community. They were analysed considering the total host population, host age and sex, as well as season and year of capture (Table 9 and Table 10). Males presented a slightly lower helminth diversity than females. Moreover, helminth diversity increases with the age of the host being similar between sub-adult and adult subpopulations. Hosts captured during spring and autumn and in 2012 presented the highest diversity, while those captured during summer and in 2013 presented the lowest.

Table 11 and Table 12 show the diversity characteristics of the helminth infracommunities, including the Brillouin index, according to host sex and age, and season and year of capture, which follow a similar trend as the helminth community diversity.

The role extrinsic (season and year of capture) and intrinsic (host age and sex) factors play in the component species of the helminth community of *M. lusitanicus* is shown in Table 13 in relation to helminth prevalence, and in Table 14 with respect to helminth abundance. The analysis of these results shows that, in general, the helminth component species of the Lusitanian pine voles were significantly affected by host age, season and year of capture. Host sex did not have any important effect on helminth prevalence and abundance.

Spearman’s correlation values between climate factors (temperature and rainfall) and diversity parameters (species richness and Brillouin index), prevalence and abundance of parasitation (Table 15) showed significantly positive correlations between prevalence and mean temperature (*T. arvicolae*) and abundance and mean temperature (*T. arvicolae* and *C. minutus*). However, the absence of any correlation between climate factors and diversity parameters stands out.

## 4. Discussion

### 4.1. Remarks on the Helminth Species

#### 4.1.1. *Hydatigera taeniaeformis* Larvae

This taenid tapeworm of cosmopolitan distribution has a diheteroxenous life cycle, having rodents as the intermediate host, with carnivores, such as felids, canids and mustelids, even domestic ones, as its definitive host [37].

#### 4.1.2. *Paranoplocephala omphalodes*

This anoplocephalan tapeworm has a Holarctic distribution with its adult stage parasitizing the small intestine of several species of the genus *Microtus*, which act as its definitive host, while some mite species act as its intermediate host [38].

#### 4.1.3. *Rodentolepis asymmetrica*

Adults of this hymenolepidid cestode parasitizes mainly the small intestine of arvicolines, also having a Holarctic distribution. Its life cycle is diheteroxenous, with some arthropod species acting as its intermediate host [34].

#### 4.1.4. *Trichuris arvicolae*

This Trichurinae nematode has been reported in some Arvicolinae species, having a Holarctic distribution, similar to their hosts. Its life cycle is monoxenous, a so-called pseudogeohelminth (the infective form for the wood mouse is not a free larva but an egg embryonating in the soil) [35,39,40].

#### 4.1.5. *Carolinensis minutus*

This heligmosomid nematode also has a Holarctic distribution and parasitizes the small intestine of some Arvicolinae species. Its life cycle is monoxenous, a so-called geohelminth (the infective form is a free larval stage, which must be ingested by the host) [36,41].

#### 4.1.6. *Heligmosomum costellatum*

This other heligmosomid nematode mainly parasitizes the small intestine of some *Microtus* species, also having a Holarctic distribution and a monoxenous geohelminth life cycle [36,42].

#### 4.1.7. *Syphacia nigeriana*

This oxyurid nematode mainly parasitizes the caecum of some Arvicolinae species, also having a Holarctic distribution. Its life cycle is monoxenous, a so-called ageohelminth, with eggs shed being infective after only a few hours.

### 4.2. Helminth Community Analysis

The helminthfauna of the *M. lusitanicus* population from Asturias shows a similar structure to other Arvicolinae, i.e., helminth species with low specificity at is host level (eurixens and oligoxens) are dominant [18,43]. Moreover, the species richness at infracommunity level is poor. The semi-fossorial life and the vegetarian diet could explain the low parasite diversity found in *M. lusitanicus* when compared to other arvicolines of the Iberian Peninsula [18,44].

Comparing the current results with previous studies [18,19,43], some differences in the helminth community composition have been found. The absence of *Notocotylus neyrai* (Trematoda), other adult cestodes, such as *Anoplocephaloides dentata* and *Arostrilepis horrida*), and *Heligmosomoides laevis* (Nematoda), as well as the presence of *R. asymmetrica* (Cestoda) and *C. minutus* (Nematoda) are the main differences observed. The lack of helminths with an indirect life cycle, which have *M. lusitanicus* as definitive host, could be explained by the use of pesticides in some crops that negatively affect the survival of intermediate hosts (arthropods and snails). Another fact to be considered is the small size of the crop fields that may increase the stochastic extinction of helminth species, while recolonization is difficult due to the poor connectivity of the habitats [45]. Indeed, a recent landscape genetics study showed that this agricultural landscape affects successful dispersal and colonization of voles at local scale [46].

The low values of helminth prevalence and mean species richness agree with the values of the diversity indices. Previous studies also obtained low prevalence [19], with *S. nigeriana* being the most prevalent helminth species. The semi-hypogea life and the herbivore diet of the Lusitanian pine vole may explain its low helminth diversity when compared to other epigean Arvicolinae from the Iberian Peninsula [18,44]. Six of the eight helminth species have an FES biological cycle, helminths which have a free-environmental infectious stage for the vole. Moreover, only five voles were parasitized after the ingestion of an infected arthropod.

The analysis of the frequency distribution of helminths revealed that dominant and co-dominant species, namely *C. minuts*, *S. nigeriana* and *H. costellatum*, showed a high level of aggregation. However, *T. arvicolae* and *H. taeniaeformis* were close to a random distribution. A high-level aggregation of parasites is associated with the stability of the host–parasite relationship, whereas low-level aggregation is associated with destabilization [47,48,49]. Consequently, according to the current results, only *T. arvicolae* and *H. taeniaeformis* might be responsible for the destabilization of the Lusitanian pine vole population in the study area.

The high aggregation of all dominant and co-dominant helminth species may be caused by various factors, such as heterogeneities in host populations and/or infection pressure [50]. The aggregation level does not seem to be affected by the helminth transmission mode or route, revealing the optimal distribution of helminths, and the co-evolutionary adaptative processes between parasites and their hosts [51]. *Hidatygera taeniaeformis* and *T. arvicolae* showed the lowest aggregation among the main helminth species found. In both cases, the infection of voles occurs after the ingestion of the egg, after its embryonation in the case of *T. arvicolae*. Both species have an FES biological cycle, the same as the other three helminth species with a high level of aggregation.

Diversity and species richness increase from younger to older hosts. According to the Kruskal–Wallis test and binary logistic regression, the species showing an age effect are *H. taeniaeformis*, *T. arvicolae* and *C. minutus.* For *H. taeniaeformis* and *T. arvicolae*, the likelihood of infection increases with age, although in *C. minutus* it decreases. In *H. taeniaeformis*, the results are the expected ones: juveniles are protected from the eggs by the IgA they ingest by the colostrum and the transplacental IgA and IgG. Adults present a partial protection from them, i.e., some eggs develop into cysts in the host liver [52]. Moreover, older animals are more likely to be infected given their age and having covered more territory to come into contact with infective forms [53]. Prevalence and intensity of helminth parasites increase with host age in other studies with wild rodents [19,54,55,56,57,58,59,60,61,62,63,64,65,66,67,68]. In *C. minutus*, the results were those to be expected. Survival of parasites decreases with age through the development of an acquired partial immunity in hosts [58]. According to the life cycle of *M. lusitanicus*, juveniles spend more time in galleries, so they are more likely to come into contact with larval forms of *C. minutus*. The acquired immunity developed against this parasite reduces the number of parasites in older individuals. Unexpected results were obtained in the case of *T. arvicolae*. This helminth is a pseudogeohelminth, whose eggs develop outside the body where they become infective forms. Younger hosts spend more time in galleries than older ones, so they have a greater likelihood to be infected. Younger animals would be expected to be more parasitized than older ones that have undergone the development of acquired immunity. However, our results showed a clear increase of infection with host age, with the absence of this helminth species in the juvenile host subpopulation.

No differences by host sex were detected in diversity and richness values. No effect in any helminth species was detected by binary logistic regression and the Mann–Whitney test. However, previous studies documented the host sex effect. Usually, males are more infected than females [69], which can be explained by different reasons [70]. Males are usually larger than females and, hence, not only provide more resources to parasites but are also more susceptible to them. Immunocompetence against infections in males is reduced as a consequence of steroid hormones, but this mechanism is not clear. Furthermore, behavioural aspects benefit infection, such as fights, reduced grooming in the mating season, covering larger territories, are all factors that increase their exposure to infective forms. However, some studies found a female bias [70]. In these cases, the reproductive state and hormone levels can affect their immunity levels and they are more susceptible to parasitic diseases. This effect increases during pregnancy and lactation. Additionally, the fact that females share nests with other females could increase the probability of infection by parasites [69]. Nevertheless, the lack of a sex bias has been reported in various small mammal species [52,59,61,68,70,71]. Some results support the idea that the influence of sex on the parasite load may depend largely on the parasite taxa and/or host-parasite associations [72,73,74,75]. Another study [70] suggests the hypothesis that a sex bias is related to a seasonally dependent sexual dichotomy in reproductive behaviour.

Analysing the effects of season, the lowest values of diversity indices are reached in summer and the highest in spring and autumn, while species richness reaches its lowest values in winter and its highest in spring. As winter is likely the season with the lowest outside activity of Lusitanian pine voles, they have fewer occasions to come into contact with parasite infective forms, which is then the reason to explain these values. According to binary logistic regression and Kruskal–Wallis test results, prevalence and abundances of *C. minutus*, *H. costellatum* and *S. nigeriana* were significantly influenced by the season of capture. *Heligmosomum costellatum* and *C. minutus* are geohelminths requiring high humidity conditions for larval survival in the environment [76]. In *H. costellatum*, higher prevalence values are linked with the reproductive stage in hosts [42]; as *M. lusitanicus* reproduces along the entire year [3], the low prevalence and intensity values in autumn are more related to environmental conditions that influence the survival of dispersal stages [77]. Summer could affect larvae survival negatively and, hence, fewer hosts are infected in autumn. So far, no data concerning the seasonal effect on *C. minutus* have been reported in the scientific literature. As with other geohelminth species, external conditions are essential for infective forms to survive. According to our results, summer and spring are the most favourable seasons for *C. minutus*. In the case of *S. nigeriana* females, when they are gravid, they migrate from the intestine to the anus. Then, they emerge and leave eggs in the perianal region, which, as an ageohelminth, become infective in a short time. These eggs can infect the same individual or other ones by grooming or sharing nests and helminths can spread among the rest of the group [77]. As *M. lusitanicus* reproduces along the year [3], the high values of *S. nigeriana* in winter could be due to the higher aggregation between individual hosts as a consequence of adverse climate conditions, spending more time in the tunnels, increasing their density and, consequently, the likelihood to be parasitized. These results agree with previous studies, which also reported positive correlations between helminth prevalence and abundances and host densities [68,78,79,80].

Concerning the year of capture, diversity index and species richness values are similar between both years, showing the medium-term stability of the Lusitanian pine vole helminth community. Among the helminth species, only the prevalence and abundance of *C. minutus* and *S. nigeriana* are influenced by the year of capture, due to the annual variability of the environmental conditions and the abundance of the host.

Although the absence of correlations between climate variables and diversity (species richness and Brillouin index) stands out, the prevalence and/or the abundance of some helminth species, such as *T. arvicolae* and *C. minutus*, with a pseudogeohelminth, respectively, and a geohelminth life cycle, correlate positively with mean temperature values of the year before capture, which influence the survival of the infective forms.

### 4.3. The Use of the Helminths of Microtus lusitanicus in Pest Control

Nowadays, the impact of parasites on reproduction and survival of wild animal populations has been recognised [81,82,83,84,85]. Moreover, parasites may play a key role in driving population regulation in vertebrates [81,86,87].

Concerning helminth species present in the helminth community of *M. lusitanicus*, Deter et al. [88,89] demonstrated that *T. arvicolae* affects the fecundity of its host, and proposed that this intestinal nematode could control population growth in several rodent species, representing an in-built pest control [90]. Likewise, it has been demonstrated that rodents infected with *H. taeniaeformis* have lower levels of leptin and higher levels of neuropeptide-Y in plasma than uninfected ones [91]. Accordingly, parasites stimulate appetite in their hosts and they become hungry. Models predict that hungry animals tend to be more willing to take risks than satiated individuals [92,93]. Therefore, infected animals as intermediate hosts are likely to be preyed upon by the final hosts of this parasite [91]. *Hydatigera taeniaeformis* larvae could play a role in population control resulting in a decrease in the number of rodents. According to our results, *T. arvicolae* and *H. taeniaeformis* might have a potential destabilization effect on the *M. lusitanicus* population, and considering their role in host population control, both species can be proposed as potential pest control in the Lusitanian pine vole population in Asturias and elsewhere in this geographic distribution.

## 5. Conclusions

The current analysis on the helminth community of the Lusitanian pine vole in Asturias provides further information on this vole pest reported in previous studies throughout its Iberian distribution, with the most remarkable difference being the near complete absence of adult cestodes parasitizing the population studied.

The influence of extrinsic factors, such as climate variables and the season and year of capture, confirms the more fragile life cycles of psedudogeohelminths, i.e., *T. arvicolae*, and geohelminths, i.e., *C. minutus*, species, while the influence of the host population density seems to affect the ageohelminth species, *S. nigeriana*, more significantly. The influence of host age was expected as it has been reported in several previous studies in various host populations, but the absence of host sex influence is surprising and remains without any convincing explanation.

An increase in the mean temperature seems to favour the transmission of these psedudogeohelminth and geohelminth species, a fact that could be of great interest considering the potential increase of mean temperatures due to global warming and climate change. Moreover, the potential destabilization effect that *H. taeniaeformis* and *T. arvicolae* could exercise on the Lusitanian pine vole population, due to their low-level aggregation, should be considered in the control programs of this studied rodent pest, even in the case of the cestode introducing its definitive hosts, carnivores which spread infective eggs and also predate the voles.

## Figures and Tables

**Table 1:** Distribution of *Microtus lusitanicus* analysed by host sex and age and season of capture.

Host Sex	Season	Host Age			
		Juveniles	Sub-Adults	Adults	Total
Males	Winter	3	26	39	68
	Spring	10	35	51	96
	Summer	6	63	36	105
	Autumn	5	63	26	94
	Total	24	187	152	363
Females	Winter	3	33	35	71
	Spring	8	38	45	91
	Summer	18	36	37	91
	Autumn	10	40	44	94
	Total	39	147	161	347

**Table 2:** Distribution of *Microtus lusitanicus* analysed by year and season of capture.

Year	Season				
	Winter	Spring	Summer	Autumn	Total
2011	25	92	110	116	343
2012	101	95	86	72	354
2013	13	0	0	0	13
Total	139	187	196	188	710

**Table 3:** Number of *Microtus lusitanicus* analysed (n) and seasonal values of climate variables corresponding to the year prior to host capture.

	n	Mean Temperature (°C)	Mean Rainfall (mm)	Cumulative Rainfall (mm)
Winter 2010	25	10.1	3.0	269.0
Spring 2010	92	15.0	4.0	338.4
Summer 2010	110	19.9	0.7	65.9
Autumn 2010	116	12.6	5.5	503.4
Winter 2011	101	11.1	2.9	246.0
Spring 2011	95	16.2	1.0	88.5
Summer 2011	86	19.7	1.3	116.2
Autumn 2011	72	14.6	2.2	199.0
Winter 2012	13	10.2	1.4	125.1

**Table 4:** Selected characteristics of the helminth community of 710 *Microtus lusitanicus* analysed.

Helminth Species	Site	LC	n	Prevalence (95% CI)	Mean Abundance (SE)	Median Intensity (Range)
CESTODA						
*Cysticercoid* sp. larvae	I	F	1	0.15 (0.1–0.5)	<0.00 (<0.00)	1 (1)
*Hydatigera taeniaeformis* larvae	L	F	59	8 (6–10)	0.10 (0.01)	1 (1–5)
*Paranoplocephala omphalodes*	I	NF	2	0.3 (0.1–0.7)	<0.00 (<0.00)	1 (1)
*Rodentolpeis asymmetrica*	I	NF	5	0.7 (0.4–2)	0.01 (<0.00)	1 (1–2)
NEMATODA						
*Trichuris arvicolae*	C	F	36	5 (4–7)	0.06 (0.01)	1 (1–4)
*Carolinensis minutus*	I	F	308	44 (39–47)	5.80 (0.91)	3 (1–417)
*Heligmosomum costellatum*	I	F	25	4 (3–6)	0.16 (0.05)	3 (1–22)
*Syphacia nigeriana*	C	F	158	22 (19–25)	4.54 (0.81)	5 (1–262)

LC, life cycle; N, number of voles parasitized; CI, confidence interval; SE, standard error; I, intestine; L, liver; C, caecum; FES, helminths which have a free-environmental infectious stage for the vole; NF, No-FES.

**Table 5:** Selected characteristics of the five most prevalent helminth species by host sex and age. CI—confidence interval, SE—standard error.

			Species				
			*H. taeniaeformis*	*T. arvicolae*	*C. minutus*	*H. costellatum*	*S. nigeriana*
Sex	Males	Prevalence (CI 95%)	9 (6–12)	3 (2–5)	21 (17–26)	2 (1–3)	13 (10–17)
		Mean Abundance (SE)	0.10 (0.02)	0.07 (0.02)	4.60 (0.93)	0.18 (0.07)	5.28 (1.22)
		Median Intensity (range)	1 (1–2)	1 (1–2)	3 (1–239)	3 (1–21)	6 (1–262)
	Females	Prevalence (CI 95%)	7 (5–10)	4 (2–6)	47 (42–52)	3 (2–5)	19 (15–23)
		Mean Abundance (SE)	0.10 (0.02)	0.06 (0.02)	7.07 (1.58)	0.13 (0.07)	3.76 (1.04)
		Median Intensity (range)	1 (1–5)	1 (1–4)	3 (1–417)	2 (1–22)	4 (1–191)
Age	Juveniles	Prevalence (CI 95%)	-	-	62 (50–74)	3 (0.4–11)	14 (7–25)
		Mean Abundance (SE)	-	-	20.19 (7.21)	0.11 (0.09)	2.51 (1.08)
		Median Intensity (range)	-	-	9 (1–417)	3.5 (2–5)	13 (1–41)
	Sub-adults	Prevalence (CI 95%)	5 (3–8)	3 (2–5)	45 (40–50)	3 (2–5)	23 (19–28)
		Mean Abundance (SE)	0.05 (0.01)	0.04 (0.01)	5.56 (1.06)	0.22 (0.10)	5.71 (1.42)
		Median Intensity (range)	1 (1–2)	1 (1–2)	3 (1–239)	4 (1–22)	4 (1–262)
	Adults	Prevalence (CI 95%)	14 (10–18)	8 (5–12)	38 (33–43)	4 (2–7)	23 (19–28)
		Mean Abundance (SE)	0.17 (0.03)	0.10 (0.02)	3.16 (0.87)	0.10 (0.04)	3.70 (1.00)
		Median Intensity (range)	1 (1–5)	1 (1–4)	2 (1–238)	1 (1–8)	5 (1–191)

**Table 6:** Selected characteristics of the five most prevalent helminth species by season and year of capture. CI—confidence interval, SE—standard error.

			Species				
			*H. taeniaeformis*	*T. arvicolae*	*C. minutus*	*H. costellatum*	*S. nigeriana*
Season	Winter	Prevalence (CI 95%)	10 (6–16)	2 (0.4–6)	28 (21-36)	6 (3–11)	30 (23–38)
		Mean Abundance (SE)	0.12 (0.03)	0.02 (0.01)	0.78 (0.21)	030 (0.17)	4.15 (1.40)
		Median Intensity (range)	1 (1–2)	1 (1)	1 (1–22)	2.5 (1–22)	3 (1–128)
	Spring	Prevalence (CI 95%)	10 (6–15)	7 (4–12)	51 (44–59)	5 (2–9)	24 (18–31)
		Mean Abundance (SE)	0.10 (0.02)	0.08 (0.02)	6.14 (0.99)	0.22 (0.12)	9.65 (2.71)
		Median Intensity (range)	1 (1)	1 (1–2)	4.5 (1–80)	2 (1–21)	11 (1–262)
	Summer	Prevalence (CI 95%)	6 (3–10)	7 (4–12)	59 (52–66)	3 (1–6)	17 (12–23)
		Mean Abundance (SE)	0.07 (0.02)	0.09 (0.03)	13.30 (3.07)	0.12 (0.06)	1.60 (0.43)
		Median Intensity (range)	1 (1–3)	1 (1–2)	5 (1–417)	4 (1–7)	4.5 (1–54)
	Autumn	Prevalence (CI 95%)	8 (5–13)	3 (1–7)	31 (25–38)	0.5 (0.1–4)	20 (15–26)
		Mean Abundance (SE)	0.11 (0.04)	0.05 (0.02)	1.36 (0.29)	0.02 (0.02)	2.80 (0.73)
		Median Intensity (range)	1 (1–5)	1 (1–4)	2 (1–31)	3 (3)	6 (1–75)
Year	2011	Prevalence (CI 95%)	8 (5–11)	5 (3–8)	51 (46–57)	3 (2–5)	16 (12–20)
		Mean Abundance (SE)	0.10 (0.02)	0.06 (0.01)	8.11 (1.64)	0.17 (0.07)	2.37 (0.61)
		Median Intensity (range)	1 (1–5)	1 (1–2)	3.5 (1–417)	4.5 (1–21)	4 (1–130)
	2012	Prevalence (CI 95%)	9 (6–12)	5 (3–8)	36 (31–41)	4 (2–7)	29 (24–34)
		Mean Abundance (SE)	0.10 (0.02)	0.07 (0.20)	3.76 (0.88)	0.15 (0.07)	6.81 (1.50)
		Median Intensity (range)	1 (1–3)	1 (1–4)	2 (1–238)	2 (1–22)	5 (1–262)
	2013	Prevalence (CI 95%)	8 (0.2–34)	-	31 (9–57)	-	-
		Mean Abundance (SE)	0.08 (0.08)	-	0.54 (0.27)	-	-
		Median Intensity (range)	1 (1)	-	1.5 (1–3)	-	-

**Table 7:** Frequency of occurrence of the number of helminth species present in infracommunities of *Microtus lusitanicus* from Asturias.

No. of Helminth Species	n	%
0	260	36.6
1	320	45.1
2	117	16.5
3	12	1.7
4	1	0.1

**Table 8:** Abundance Index (AI) and Lefkovitch Index (L) of the most prevalent helminth species of *Microtus lusitanicus* from Asturias.

Helminth Species	AI	L
*Hydatigera taeniaeformis* larvae	-	0.23
*Trichuris arvicolae*	0.06	0.24
*Carolinensis minutus*	5.80	0.99
*Heligmosomum costellatum*	0.16	0.88
*Syphacia nigeriana*	4.54	0.99

**Table 9:** Diversity characteristics of the helminth community of *Microtus lusitanicus* from Asturias by host sex and age.

	Sex		Age			
	Males	Females	Juveniles	Sub-Adults	Adults	Total
Shannon index	0.86	0.78	0.38	0.82	0.93	0.84
Simpson index	0.53	0.48	0.20	0.53	0.55	0.52
Berger–Parker index	0.48	0.36	0.11	0.51	0.49	0.46
Shannon evenness index	0.44	0.40	0.34	0.46	0.45	0.40

**Table 10:** Diversity characteristics of the helminth community of *Microtus lusitanicus* from Asturias by season and year of capture.

	Season				Year		
	Winter	Spring	Summer	Autumn	2011	2012	2013
Shannon index	0.76	0.81	0.45	0.81	0.68	0.81	0.38
Simpson index	0.38	0.50	0.22	0.48	0.39	0.49	0.22
Berger–Parker index	0.23	0.41	0.12	0.35	0.25	0.38	0.13
Shannon evenness index	0.39	0.41	0.25	0.50	0.38	0.39	0.54

**Table 11:** Diversity characteristics of helminth infracommunities of *Microtus lusitanicus* (*M.l.*) from Asturias by host sex and age. SE—standard error.

		Sex		Age			
		Male	Female	Juveniles	Sub-Adults	Adults	Total
Mean species richness	X	0.86	0.81	0.79	0.80	0.88	0.84
	SE	0.04	0.04	0.08	0.04	0.05	0.03
Brillouin index		0.06	0.06	0.05	0.06	0.07	0.06
	SE	0.01	0.01	0.02	0.01	0.01	0.01
	Max	0.60	0.75	0.62	0.65	0.75	0.75
BI infected *M.l.* only	X	0.07	0.06	0.04	0.06	0.06	0.06
	SE	0.01	0.01	0.02	0.01	0.01	0.01
% of *M.l.* infected		62.5	64.3	68.3	61.1	64.9	63.38

**Table 12:** Diversity characteristics of helminth infracommunities of *Microtus lusitanicus* (*M.l.*) from Asturias by season and year of capture. SE—standard error.

		Season				Year		
		Winter	Spring	Summer	Autumn	2011	2012	2013
Mean species richness	X	0.78	1.01	0.92	0.62	0.83	0.86	0.38
	SE	0.07	0.06	0.05	0.05	0.04	0.04	0.14
Brillouin index	X	0.06	0.07	0.05	0.05	0.06	0.06	0.02
	SE	0.01	0.01	0.01	0.01	0.01	0.01	0.02
	Max	0.65	0.62	0.55	0.75	0.75	0.65	0.28
BI infected *M.l.* only	X	0.07	0.08	0.06	0.04	0.06	0.06	0.06
	SE	0.02	0.01	0.01	0.01	0.01	0.01	0.01
% of *M.l.* infected		56.8	73.8	71.4	49.5	65.0	62.7	38.5

**Table 13:** Binary logistic regression models for the prevalence of the helminth component species of *Microtus lusitanicus* from Asturias by year and season of capture, host sex and age, expressed by *χ^2^* values with associated probabilities (*p*) for the model created, including independent variables. Only statistically significant models are reported.

Helminth Species/Independent Variables Included in the Model	df	*χ* ^2^	*p*
Global *M. lusitanicus* parasitation			
Season of capture	3	32.471	<0.0001
*Hydatigera taeniaeformis* larvae			
Host age	2	27.540	<0.0001
*Trichuris arvicolae*			
Host age	2	13.789	0.001
*Carolinensis minutus*			
Host age	2	13.333	0.001
Host age and sex	2	7.189	0.027
Season of capture	3	49.574	<0.0001
Year of capture	2	17.216	<0.0001
*Heligmosomum costellatum*			
Season of capture	3	11.074	0.011
*Syphacia nigeriana*			
Season of capture	3	8.711	0.033
Year of capture	2	23.797	<0.0001

Df—degree of freedom.

**Table 14:** Values of Mann–Whitney (U) and Kruskal–Wallis (χ^2^) tests, with associated probabilities (*p*), applied in the analyses of the helminth component species abundances of *Microtus lusitanicus* from Asturias. The Mann–Whitney test was applied to host sex and the Kruskal–Wallis test was applied to host age, season and year of capture. Only significant results are reported.

Helminth Species/Independent Variables Included in the Model	df	U/χ^2^	*p*
*Hydatigera taeniaeformis* larvae			
Host age	2	23.327	<0.0001
*Trichuris arvicolae*			
Host age	2	10.992	0.004
*Carolinensis minutus*			
Host age	2	23.558	<0.0001
Season of capture	3	68.329	<0.0001
Year of capture	2	21.101	<0.0001
*Heligmosomum costellatum*			
Season of capture	3	8.868	0.031
*Syphacia nigeriana*			
Season of capture	3	8.386	0.039
Year of capture	2	20.706	<0.0001

df—degree of freedom.

**Table 15:** Values of Spearman Correlation Coefficient (*r*_s_), with associated probabilities (*p*), between climate variables (temperature and rainfall) and diversity (species richness and Brillouin index), prevalence and abundance. Only significant results are reported.

Correlations	*r*_s_	*p*
*Trichuris arvicolae* prevalence and mean temperature	0.691	0.009
*Trichuris arvicolae* abundance and mean temperature	0.663	0.014
*Carolinensis minutus* abundance and mean temperature	0.698	0.008

## Data Availability

The database used to carry out the present study is not publicly available due to internal policy of our departments. However, the database could be available, after a justification of its use, upon request from the corresponding author.
